# Spacial Energy Distribution Manipulation with Multi-focus Huygens Metamirror

**DOI:** 10.1038/s41598-017-09474-w

**Published:** 2017-08-22

**Authors:** Zhuochao Wang, Xumin Ding, Kuang Zhang, Qun Wu

**Affiliations:** Department of Microwave Engineering, Harbin Institute of Technology, Harbin, 150001 China

## Abstract

Huygens metasurface is a planar array of crossed electric and magnetic dipoles, which provide specific surface current to tailor the electromagnetic field distribution. By changing the geometrical parameters of the proposed unit cell, the manipulation range of reflection phase can achieve 2π, while the amplitude of the reflection coefficient can keep above 0.993. Based on the designed Huygens meta-atoms, a novel multi-focus Huygens metamirror is proposed at microwave range in this paper. Utilizing the meta-atoms with the desired reflection phase distribution as calculated, the incident plane wave can be converged to designated points in any desired fashion including focal number, location and intensity distribution, which exhibits outstanding manipulation capability. Our research on Huygens metamirror provides a fascinating design of multi-focus imaging in microwave region, which makes it potential applications in antenna and imaging systems.

## Introduction

Metasurface, a novel two-dimensional metamaterial composed of artificially designable meta-atoms, has attracted considerable attention due to its outstanding ability of electromagnetic manipulation with subwavelength thickness. Constantly emerging papers in this field have promoted the flourish of correlative researches, including beam shaping lens^1–3^, polarization transformer^4, 5^, directional radiator^6, 7^, thin-film cloaking^8, 9^, imaging hologram^10–12^ and other potential applications. However, ultrathin metasurface with the ability to control the amplitude and phase of the transmission and reflection coefficient completely is still a great challenge in metasurface design.

The presence of Huygens metasurface provides a systematic theoretical foundation to achieve the manipulation of transmission and reflection independently. Magnetic and electric dipoles are collocated into each Huygens meta-atom to generalize specific electric and magnetic surface response under the incidence. Then electromagnetic field distribution can be tuned arbitrarily. Due to the fascinating wave-manipulating ability of Huygens metasurface, various applications have been reported in beam-refracting^13–16^, focusing^17, 18^, cloaking^9, 19^, beam-shaping^20, 21^ and polarization control^22, 23^. All metasurfaces mentioned above exhibit reflectionless property. Here we propose a lossless reflective Huygens metasurface to realize multi-focus metamirrors. By elaborately designing the geometrical shape of the magnetic and electric dipoles, the metasurface can provide a 2π range of reflection phase, while the amplitude of the reflection coefficient can keep above 0.993 according to the full-wave simulation results. Multi-focus Huygens metamirrors are designed to converge the incident beam to specified positions at microwave range, as an application of the meta-atom model. To control the reflected field distribution completely, the formulas of the reflection phase for each meta-atom is proposed here based on the process of hologram^24^. As the formulas illuminated, arbitrary multi-focus imaging can be realized. Compared with traditional focusing metamirrors, besides the focal number and location, the focal intensity distribution can also be manipulated. Here single-focus metamirrors and double-focus metamirrors with specific focal intensity ratio are designed individually. Our numerical simulation results of the proposed metamirrors agree very well with the theoretical results, which adds a new degree of freedom to the manipulation of electromagnetic wave.

## Results

### Reflective Huygens Metasurface Design

Huygens metasurface is a two-dimensional array composed of crossed magnetic and electric dipoles which can generate specific electromagnetic response under normal incidence. The electromagnetic response can be produced by the induced surface electric and magnetic current, which can manipulate the scattered electric and magnetic field distribution above and below the metasurface indirectly according to the boundary condition. Surface electric and magnetic impedance are defined here to build the relationship between the surface current and the electromagnetic field. It can be modified by changing the structure of meta-atom appropriately. Then, based on the scattered field distribution, the relationship between the reflection and transmission coefficient and the surface impendence can be built. Overall, according to the equivalence principle and boundary conditions, arbitrary reflection and transmission coefficient can be realized with specific surface electromagnetic impedance^25, 26^.

1 $${Z}_{e}=\frac{\eta }{2}\frac{1+(R+T)}{1-(R+T)}$$

2 $${Z}_{m}=2\eta \frac{1+(R-T)}{1-(R-T)}$$

where $R=r{e}^{j{\varphi }_{r}}$ and $T=t{e}^{j{\varphi }_{t}}$ are the predetermined reflection and transmission coefficients. *Z*_*e*_ and *Z*_*m*_ denotes surface electric and magnetic impedance respectively. For the design of reflective Huygens metasurface, the reflection coefficient should remain to be 1 while the transmission coefficient equals 0. Therefore, when *r* = 1 and *t* = 0, equations (1) and (2) become

3 $${Z}_{e}=j\frac{\eta }{2}\frac{sin{\varphi }_{r}}{2-2cos{\varphi }_{r}}$$

4 $${Z}_{m}=j2\eta \frac{sin{\varphi }_{r}}{2-2cos{\varphi }_{r}}$$

where *φ*_*r*_ represents the reflection phase, which changes from 0 to 2π. *η* is set to be 377Ω as the impedance of the free space. From the above two expressions, *Z*_*e*_ and *Z*_*m*_ are pure imaginary, so the reflection phase can be controlled only by a lossless metasurface theoretically.

**Figure 1:**
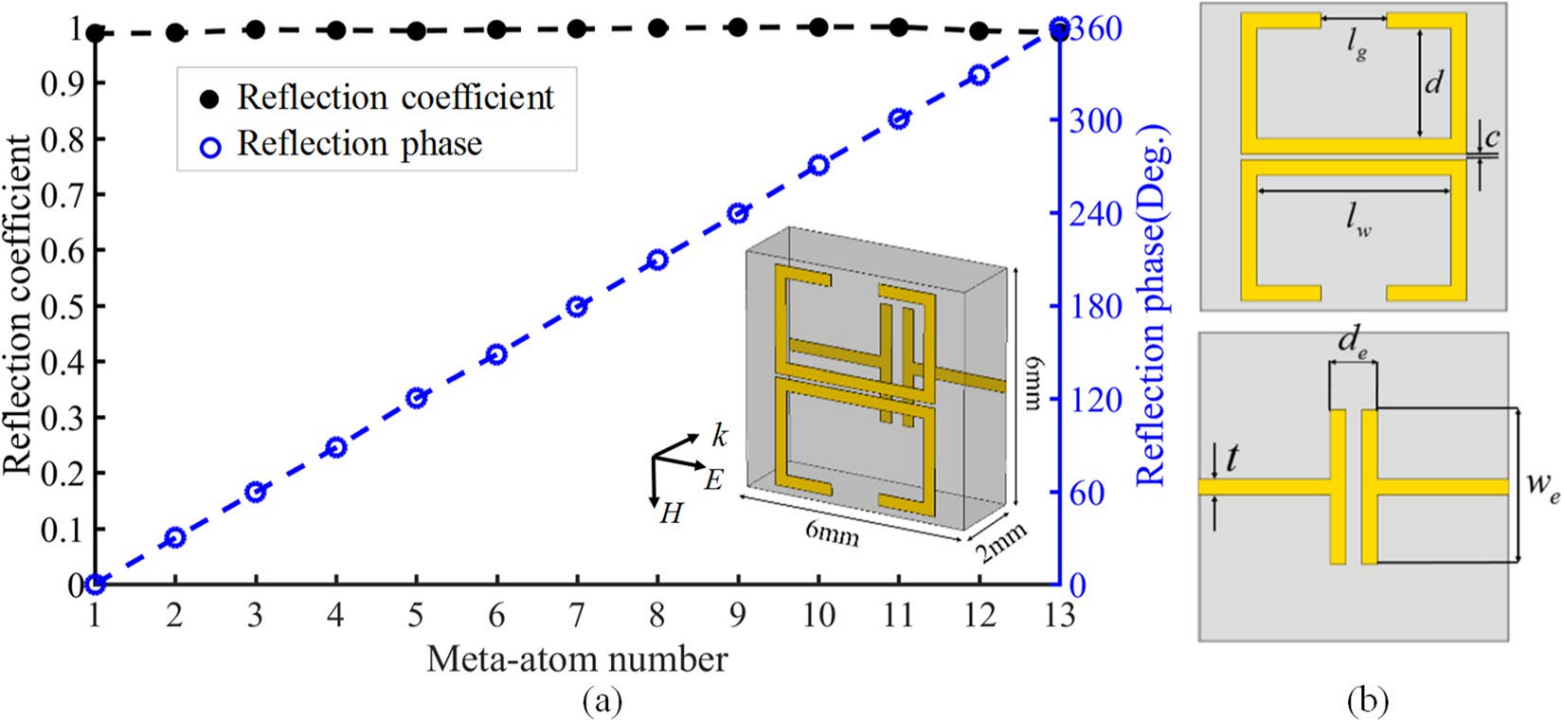
Schematic diagram and reflection characteristics of the proposed Huygens meta-atom. (**a**) Simulation results of reflection coefficients and reflection phase shift for meta-atom with different parameters. (**b**) Geometric parameters of the front view and back view of the meta-atom.

**Figure 2:**
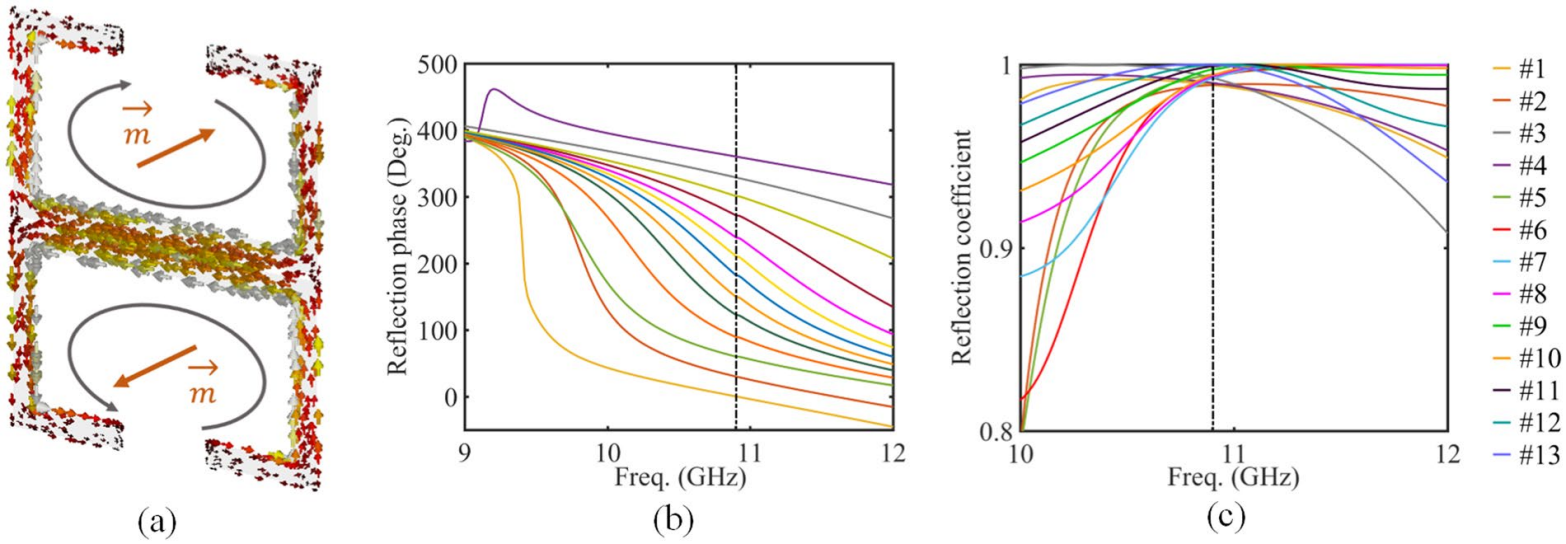
(**a**) Schematic diagram of particular design principle about the magnetic response (**b**) The simulated reflection phase of meta-atoms to prove the manipulation range of 2*π*. (**c**) The simulated reflection coefficient of meta-atoms to prove the high efficiency.

The desired electric and magnetic impendence can be achieved by the proposed meta-atom, as shown in Fig. 1. Two mirror-symmetry metal split rings are placed on the top layer of the substrate, operated as the magnetic dipoles. By tuning the arm length of the magnetic dipoles, marked as *l*_*w*_ and *l*_*g*_, the magnetic response can be manipulated. Meanwhile, the electric dipole is composed of two mirror-symmetry T-shaped metal stripes and the induced electric current can be modified by changing the capacitor width *w*_*e*_. The magnetic and electric current should be mutually perpendicular, so the generated electromagnetic field can be manipulated individually without interference. The proposed unit cell is simulated using the commercial software, CST Microwave Studio, to analysis the feature of the proposed meta-atom. Meanwhile, the incidence is set as normal TM microwave to ensure the electric field parallel to electric dipole. According to simulation results, it works at 10.9 GHz. Figure 1(b) shows the designed meta-atom, which composed of metal stripes integrated on dielectric substrate. Except the variable parameters mentioned above, the other parameters remain to be constant, as *d* = 2.45 *mm*, *c* = 0.1 *mm*, *d*_e_ = 0.9 *mm*, *t* = 0.3 *mm*. Compared with previous researches, the proposed Huygens meta-atom composed of double mirror-symmetric magnetic dipoles, instead of the single one. This mirroring effect can support toroidal dipole excitation. As shown in Fig. 2(a), the simulated surface current along two metal split rings circles in opposite directions. Therefore, two induced magnetic dipole ($\overrightarrow{m}$) are head-to-tail in a small circular region, which generates the toroidal dipole^27^. There are two merits of this modification. First, since the quality factor of toroidal resonance is higher than the magnetic resonance, full manipulation of the reflection phase can be achieved by changing the arm length, as shown in Fig. 2(b). Therefore, there is no need to change the shape of metal patch significantly, which simplifies the design of meta-atom. Second, the mirror-symmetric structure guarantees the direction of magnetic dipole opposite with neighboring one. This design can dramatically reduce the radiation loss and improve the efficiency^28^. Figure 1(a) illustrates the simulation results of the proposed Huygens meta-atom. The range of reflection phase can cover the whole range of 360 degrees and the step of phase shift is set to be 30 degrees. Actually, arbitrary phase shift can be realized with accurate geometric design. Meanwhile, the amplitude of the reflection coefficient keeps above 0.993 and the amplitude of the transmission coefficient keeps below 0.154, which means the energy of the incidence can be reflected and controlled totally by our design.

## Multi-focus Metamirrors

Multi-focus imaging can be achieved by modulating phase distribution of the metasurface. By introducing desired phase changes to the reflected wave utilizing the proposed meta-atom, the incident wave can be focused to specified positions. One of the fundamental methods of designing hologram is setting virtual point sources at the preset focal locations^29, 30^. Then the electric field generated by each virtual source can be superimposed to obtain the virtual electric field distribution. Here the electromagnetic field propagation from each virtual point source is described by Green function. Then the virtual electric field is mapped onto the metasurface and the desired phase delay at the positions of different meta-atoms *φ*(*x*, *y*, 0) can be calculated. To perform the desired focusing image, the reflection phase of each meta-atom should be set as −*φ*(*x*, *y*, 0) under the incident plane wave^24^, since the calculation of phase conjugation in frequency domain is equivalent to the time reversal for the incident monochromatic wave^31, 32^. Accordingly, the reconstructed electric field is converged to the positions where virtual sources locate. However, the electric field intensity decreases linearly with the reciprocal of the distance between meta-atoms and virtual sources. The calculation of phase conjugation only ensures the incident wave focus at preset locations, but can’t eliminate the difference of intensity between the focal points. Therefore, the method mentioned above is only applicable to the situation of single focus. For multiple-focus imaging, the intensity of virtual point sources should be modified with different weight based on the specific focal intensity distribution. Hence, the assigned reflection phase of each meta-atom is modified as

5 $$-\varphi ({x}_{j},{y}_{j},0)=-{\rm{\arg }}(\sum _{i=1}^{N}{w}_{i}G({r}_{mi}))=-arg(\sum _{i=1}^{N}{w}_{i}\frac{{e}^{-jk{r}_{mi}}}{4\pi {r}_{mi}})(j=1\,to\,M)$$

where ${r}_{mi}=\sqrt{{({x}_{j}-{x}_{i})}^{2}+{({y}_{j}-{y}_{i})}^{2}+{{z}_{i}}^{2}}$ is the distance between the *j* ^*th*^ meta-atom located at (*x*_*j*_, *y*_*j*_, 0)(*j* = 1 *to M*) and the *i* ^*th*^ focal point at (*x*_*i*_, *y*_*i*_, *z*_*i*_)(*i* = 1 *to N*). *k* is the phase constant. *w*_*i*_ is the weight factor of *i* ^*th*^ virtual source, which presents the intensity ratio of the *i* ^*th*^ virtual source to the first one. Particularly, *w*_1_ is set to 1. Then, the reflection phase is determined to be −*φ*(*x*_*j*_, *y*_*j*_, 0) for the meta-atom located at (*x*_*j*_, *y*_*j*_, 0).

Next, based on the established reflection phase distribution, the reconstructed electric field distribution can be calculated through the superposition of the electric field component generated by each meta-atom. Since the electric field component generated by each meta-atom can be described by the Green function theoretically, the reconstructed electric field distribution under the incident plane wave is described as

6 $$E(x,y,z)=\sum _{j=1}^{M}{e}^{jk\varphi ({x}_{j},{y}_{j},0)}G({r}_{mj})=\sum _{j=1}^{M}\frac{{e}^{j(arg({\sum }_{i=1}^{n}{w}_{i}\frac{{e}^{-jk{r}_{mi}}}{4\pi {r}_{mi}})-k{r}_{mj})}}{4\pi {r}_{mj}}$$

where ${r}_{mj}=\sqrt{{(x-{x}_{j})}^{2}+{(y-{y}_{j})}^{2}+{z}^{2}}$ denotes the distance between the meta-atom at (*x*_*j*_, *y*_*j*_, 0) and the observation point at (*x*, *y*, *z*). Here the observation point is set at (*x*_*i*_, *y*_*i*_, *z*_*i*_) to get the intensity of *i* ^*th*^ reconstructed focal point, |*E*(*x*_*i*_, *y*_*i*_, *z*_*i*_)|, denoted by the amplitude of the electric field at the position of the *i* ^*th*^ preset focal point.

Then, the relationship between *w*_*i*_ and the intensity distribution of focal points is analyzed to achieve the desired imaging. To describe the intensity distribution of focal points, the intensity ratio of the *i* ^*th*^ focal point to the first one is defined as *k*_*i*_. Based on the equation (5), *w*_*i*_ is the weight factor of intensity of the *i* ^*th*^ virtual focal point, which can directly affect the intensity distribution of reconstructed electric field. Particularly, *w*_*i*_ doesn’t equal to *k*_*i*_ here, since the reflection coefficient of each meta-atom on the surface keeps the same, and the only variable parameter is the reflection phase. Therefore, the virtual electric field differs from the reconstructed one slightly, which means it is not a completely reversible process. Hence, *w*_*i*_ is modified to guarantee the reconstructed electric field at focal points equal to the desired intensity ratio (*k*_*i*_). Accordingly, the formulation is described as

7 $$\frac{|E({x}_{i},{y}_{i},{z}_{i})|}{|E({x}_{1},{y}_{1},{z}_{1})|}={k}_{i}\,(i=2\,to\,N).$$

**Figure 3:**
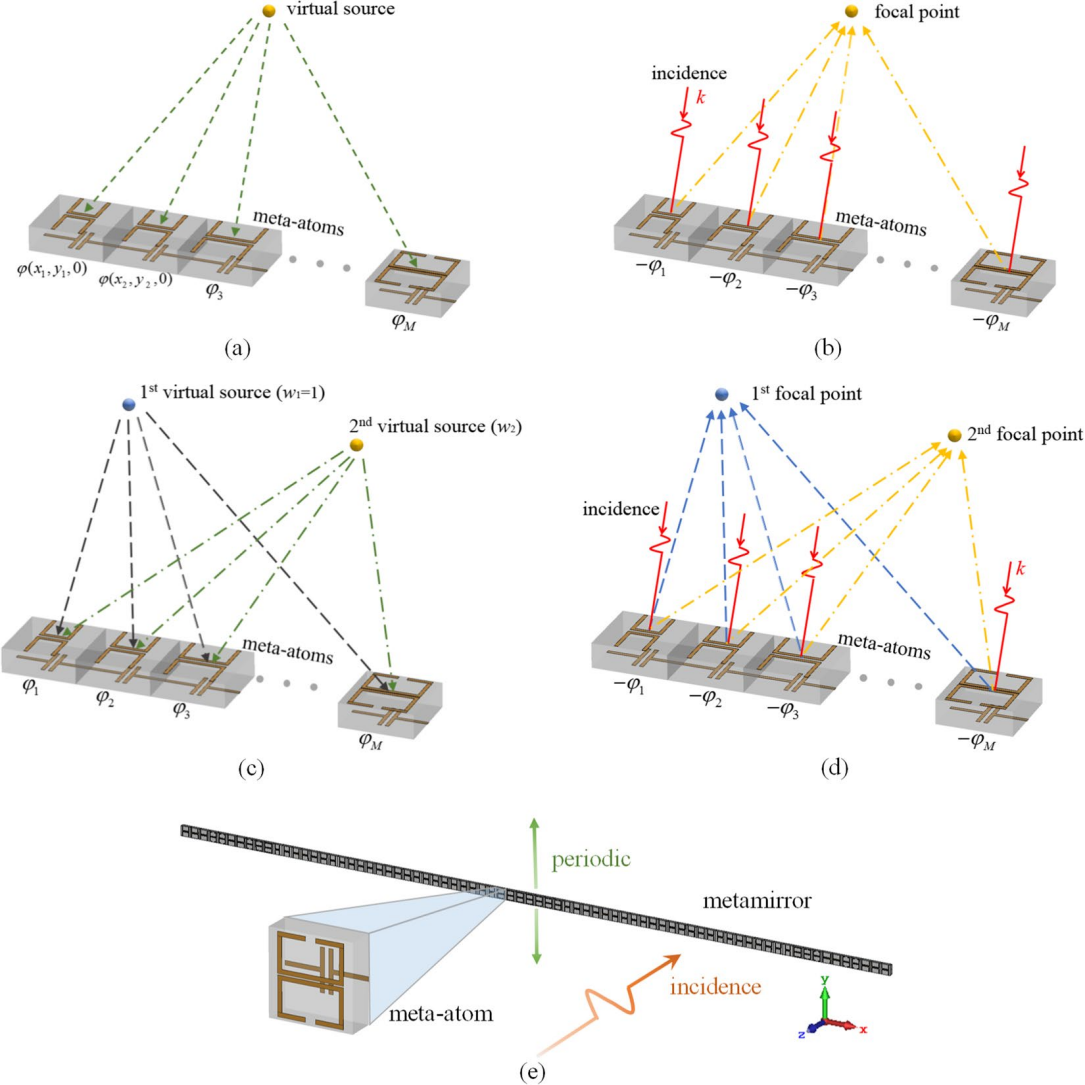
Schematic diagram of design process. (**a**) (**b**) The virtual and reconstructed electric field for single-focus metamirror. (**c**), (**d**) The virtual and reconstructed electric field for double-focus metamirror. (**e**) The structure of metamirror.

For application of the proposed algorithm, we design single-focus and double-focus metamirrors with specific energy intensity distribution respectively. For the single-focus metamirror, the first step is to set a virtual source at the position of focal point to get the phase delay (*ϕ*_1_, *ϕ*_2_ …, *ϕ*_*M*_) on the metasurface, as shown in Fig. 3(a). Next, as Fig. 3(b) illustrates, each meta-atom is assigned negative reflection phase delay (−*ϕ*_1_, −*ϕ*_2_ …, −*ϕ*_*M*_) to ensure the electric field emitted from each meta-atom superimposes in phase at the position of foal point.

When the number of focal points exceeds one, using our proposed algorithm, specific focal intensity ratio of the focal points can be realized. As shown in Fig. 3(c), the virtual electric field generated from double virtual sources with the intensity ratio (*w*_*i*_) is superposed to calculate the phase delay onto each meta-atom (*ϕ*_1_, *ϕ*_2_ …, *ϕ*_*M*_). Similarly, the reflection phase distribution on the metasurface is set to the negative phase delay (−*ϕ*_1_, −*ϕ*_2_ …, −*ϕ*_*M*_), as shown in Fig. 3(d). This operation is to divert the energy to the desired focal points. Furthermore, by determining the value of *w*_2_ according to the equation (7), the intensity ratio of the reconstructed electric field at two focal points can equal to desired *k*_2_. Therefore, the manipulation of the intensity distribution between the focal points can also be achieved utilizing the phase change of each meta-atom.

To demonstrate our theoretical analysis, two types of metamirrors have been simulated with the commercial simulation software FDTD Solutions respectively. Here meta-atoms change along the *x*-axis to achieve the desired reflection phase distribution, while the meta-atoms keep the same along the *y*-axis. In our simulations, the boundary condition along *y*-direction is set to be the perfect electric conductor (PEC) boundary. Hence, the focusing performance remains the same along the *y*-axis, and *xoz* plane reflects the focusing features of the designed metamirror, as shown in Fig. 3(e).

**Figure 4:**
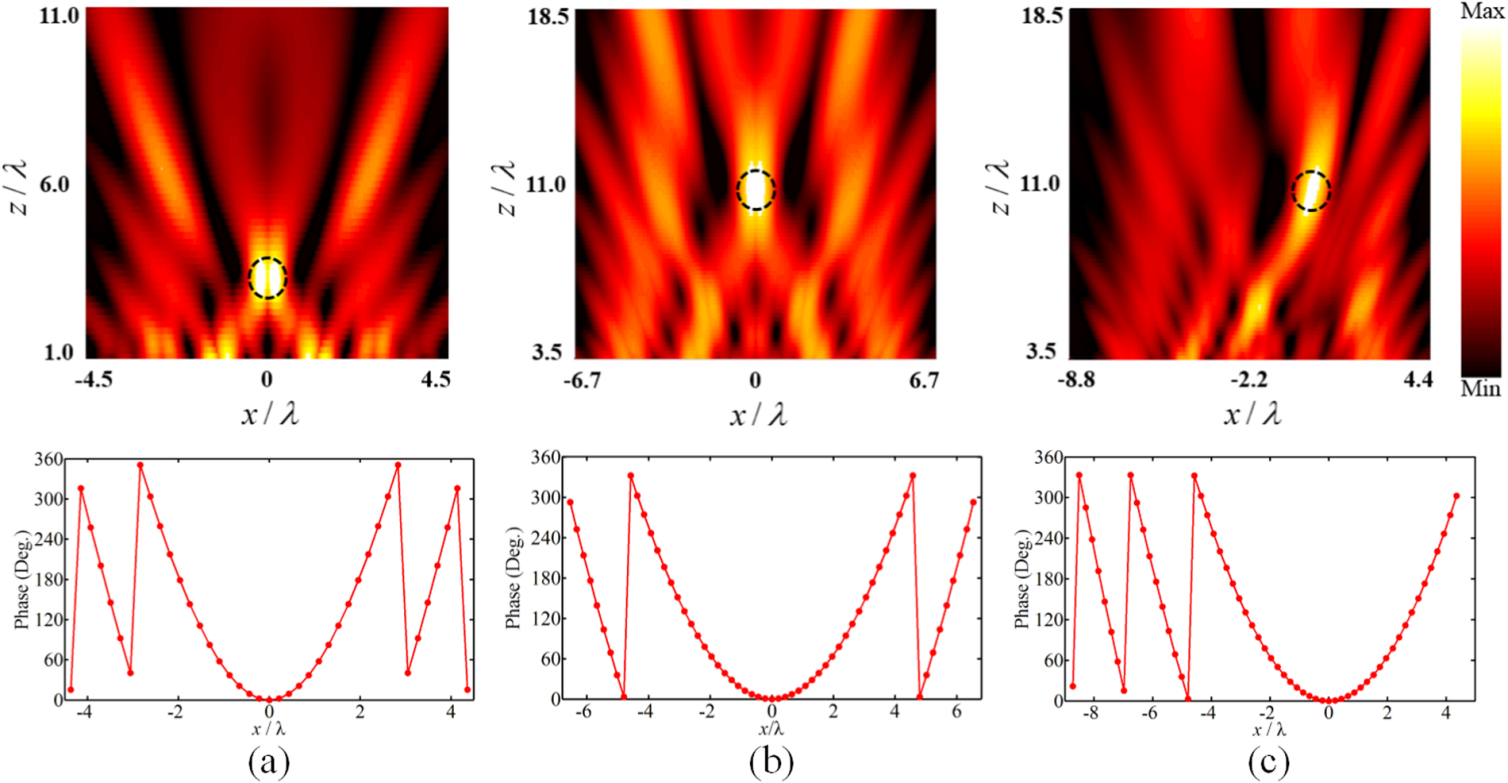
Simulation results of electric field distribution and phase distribution of metasurface for single-focus metamirrors. The incidence wave is converged to three specific points located at (**a**) *x* = 0, *z* = 3.5*λ*, (**b**) *x* = 0, *z* = 11*λ*, and (**c**) *x* = 1*λ*, *z* = 11*λ* respectively.

Firstly, we design single-focus metamirrors to converge the incident plane wave to desired focal points in *xoz* plane. Figure 4 shows the electric field distribution in *xoz* plane and the required spatial phase of each meta-atom. The simulation results show the single focal point can be moved along x and z direction at will. It demonstrates that the incidence can be converged to any single point in *xoz* plane with calculated phase distribution described by equation (5).

**Figure 5:**
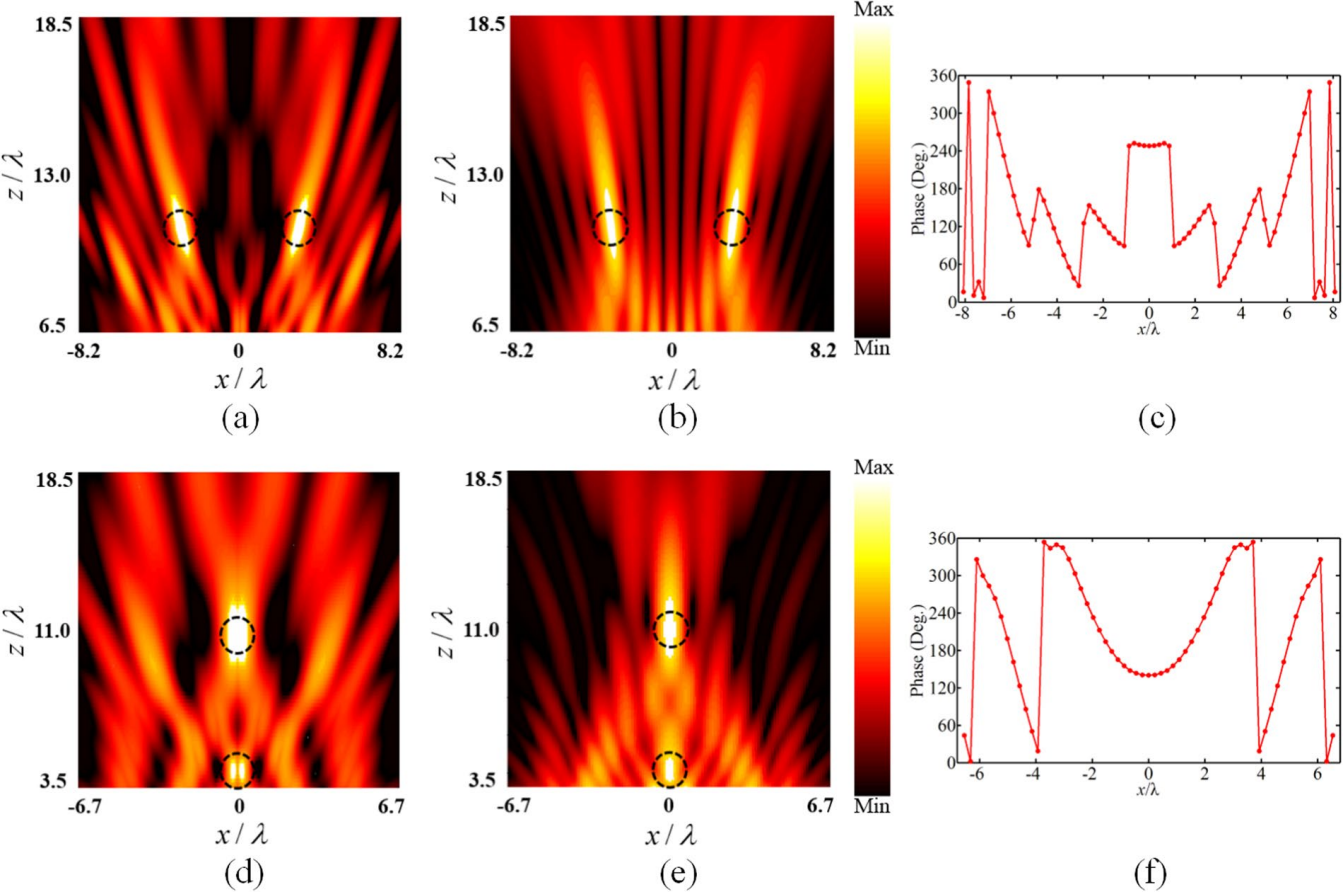
Design of double-focus metamirrors with uniform focal intensity distribution. (**a**), (**b**) Numerical simulations (left column) and theoretical results (middle column) of electric field intensity distribution with two foci located at *x* = −3.3*λ*, *z* = 11*λ* and *x* = 3.3*λ*, *z* = 11*λ*. (**c**) Corresponding reflection phase shift distribution on the chain of 61 meta-atoms for two foci symmetric about *x*-axis. (**d**), (**e**) Numerical simulations (left column) and theoretical results (middle column) of electric field intensity distribution with two foci located at *x* = 0, *z* = 3.5*λ* and *x* = 0, *z* = 11*λ*. (**f**) Assigned reflection phase shift distribution on the chain of 71 meta-atoms for two foci symmetric about *z*-direction.

Different from the design of single-focus metamirrors, the weight factor needs to be taken into consider for double-focus imaging. Figure 5 illustrates the design of two focal points with the same intensity placed along x-axis and z-axis respectively. To achieve the uniform intensity ratio, the weight factor is modified to compensate the decrease of electric field intensity. As shown in Fig. 5, the simulation results of the electric field distribution agree very well with the theoretical ones. Furthermore, the corresponding phase distribution of the metasurface is shown in the right column.

**Figure 6:**
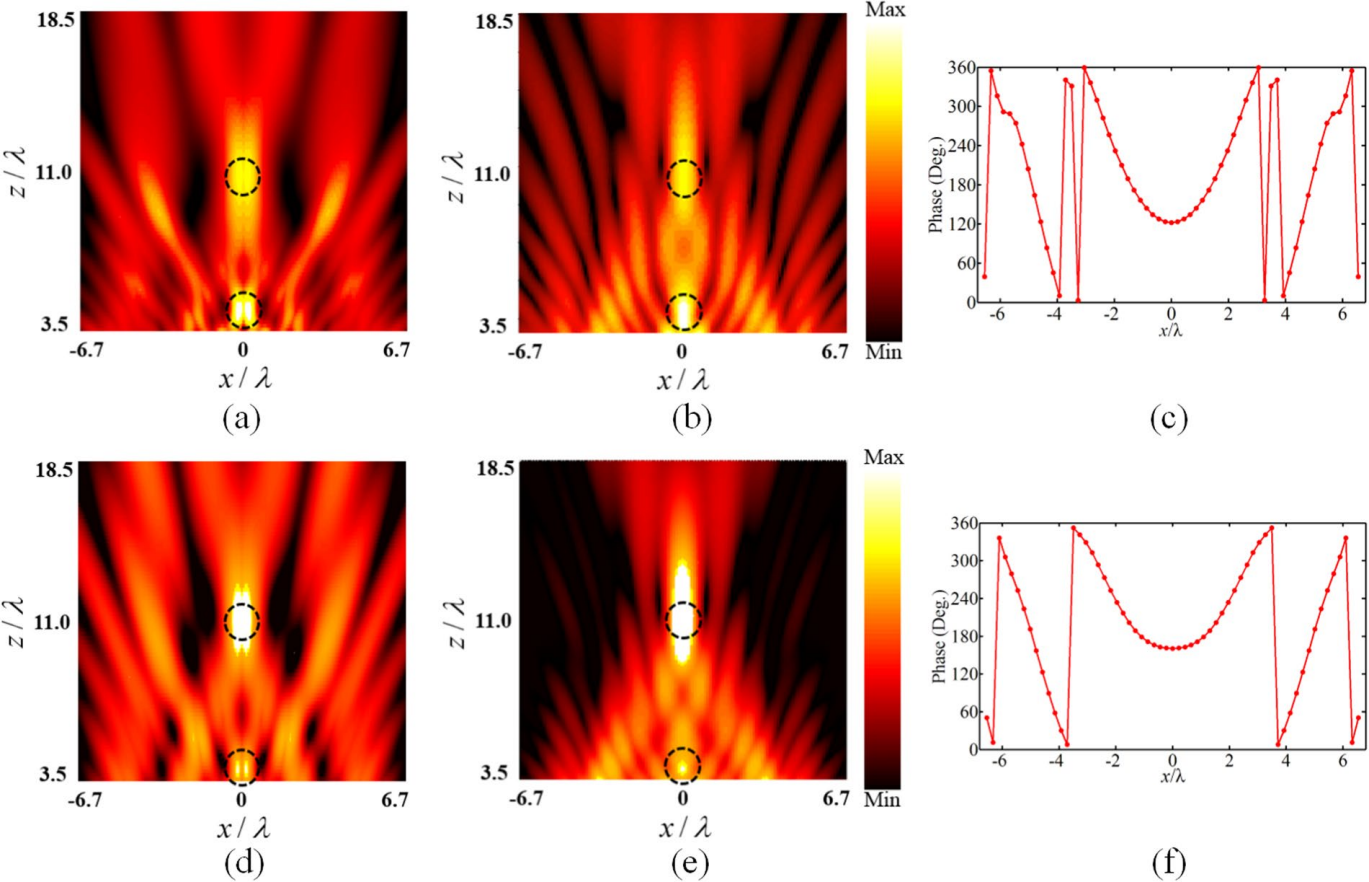
Design of metamirrors of two foci located at *x* = 0, *z* = 3.5*λ* and *x* = 0, *z* = 11*λ* with specific intensity ratio. (**a**), (**b**) Numerical simulations (left column) and theoretical results (middle column) of electric field intensity distribution with the focal intensity ratio *k*_2_ = 0.8 and the weight factor *w*_1_ = 1, *w*_2_ = 3.8. (**c**) Corresponding reflection phase shift distribution on the chain of 61 meta-atoms with higher intensity of focus at *z* = 3.5*λ*. (**d**), (**e**) Numerical simulations (left column) and theoretical results (middle column) of electric field intensity distribution with the focal intensity ratio *k*_2_ = 1.3 and the weight factor *w*_1_ = 1, *w*_2_ = 10. (**f**) Assigned reflection phase shift distribution on the chain of 61 meta-atoms with higher intensity of focus at *z* = 11*λ*.

Besides the uniform intensity ratio, by changing the weight factor, we can also achieve arbitrary focal intensity distribution. Figure 6 illustrates the design of two focal points ranged along *z*-axis with different focal intensity ratio. In both situations, the incident plane wave is converged to the desired focal location, and the simulation results of focal intensity ratio are in good agreement with the theoretical ones. Hence, it indicates that the weight factor can manipulate the intensity ratio of each focal point respectively, adding a new degree of freedom to the manipulation of electromagnetic wave.

## Discussion

In conclusion, lossless reflective Huygens metasurface composed of a series of crossed electric and magnetic dipoles are designed and simulated in this paper. The reflection phase of the proposed meta-atoms can be manipulated in the whole range of 2π with high efficiency. Based on them, multi-focus Huygens metamirrors have been first proposed. Arbitrary focus imaging can be achieved at microwave range in consideration of the factors of focal number, location and intensity ratio. To demonstrate it, the intensity distribution of the electric field on the focal plane is simulated, which agrees very well with the theoretical analysis. The proposed multi-focus Huygens metamirrors can be applied to many fields, which makes our design a potential application in antenna and imaging systems. Furthermore, the design method of reflective Huygens metasurface with the function of multi-focusing proposed in this paper can be applied to other frequency ranges.
